# COMPARISON BETWEEN PORTALS FOR PLACEMENT OF ANCHORS IN SHOULDER INSTABILITY

**DOI:** 10.1590/1413-785220243201e265443

**Published:** 2024-05-06

**Authors:** José Carlos Souza Vilela, Ivana Duval de Araújo, Mabelly Correa Albuquerque, Anna Luiza Amancio Vidal, Tadeu Fonseca Barbosa, Thalles Leandro Abreu Machado

**Affiliations:** 1. Hospital Unimed BH, Serviço de Ortopedia e Traumatologia, Belo Horizonte, MG, Brazil.

**Keywords:** Shoulder Dislocation, Arthroscopy, Joint Instability, Luxação do Ombro, Artroscopia, Instabilidade Articular

## Abstract

**Objective:**

*to radiographically compare the effects of anchor positioning in the arthroscopic treatment of shoulder instability, in the 3- and 5-o’clock portals.*

**Methods:**

*retrospective study of 36 patients, operated by two shoulder surgeons at the Unimed BH hospital, between January 2013 and January 2018. Each surgeon used only one of either the 3- or the 5-o’clock portal. After postoperative radiographs we performed angle comparisons between the greatest glenoidal axis, the angle of anchor insertion and distance from the inferior pole.*

**Results:**

*the 5-o’clock portal provided better placement than its 3-o’clock counterpart, which allowed for greater orthogonality in relation to the glenoid rim (p < 0.05).*

**Conclusion:**

*the 5-o’clock portal allowed for better anchor placement than the 3 o’clock one.* ***Level of Evidence II, Clinical Trial.***

## RESUMO

**Objetivo:**

*Comparar radiograficamente o posicionamento das âncoras utilizadas no tratamento artroscópico da instabilidade do ombro, através dos portais de 3 ou 5 horas.*

**Métodos:**

*Avaliação retrospectivae de 36 pacientes, operados por dois cirurgiões de ombro do Hospital Unimed BH, entre janeiro de 2013 e janeiro de 2018. Cada cirurgião utilizou apenas uma das técnicas – portal de 3 ou 5 horas. As radiografias pós-operatórias foram avaliadas e comparadas a angulações entre o maior eixo da glenoide, o ângulo de inserção da âncora e a distância em relação ao polo inferior.*

**Resultados:**

*A utilização do portal de 5 horas propiciou a colocação mais adequada das âncoras em relação ao portal de 3 horas, permitindo o posicionamento mais ortogonal em relação à borda da glenoide (p < 0,05).*

**Conclusão:**

*A utilização do portal de 5 horas apresenta melhor posicionamento das âncoras quando comparado ao portal de 3 horas.* ***Nível de evidência II, Ensaio Clínico.***

## INTRODUCTION

 Capsulolabral reinsertion of the glenoid was first described by Bankart ^1^ for the treatment of anterior shoulder dislocation. Open repair with curved needles used to be the standard; nowadays, though, the modernization of techniques and surgical materials led to a gradual presence of arthroscopy in surgical treatment of shoulder instability. 

 Traditionally, anterior anchors in the glenoid are placed through the anteroinferior portal (3-o’clock position) which provides optimal access to the anterior face of the glenoid; however, it is sometimes difficult to place the anchor in the most inferior face of the glenoid due to the acuteness of the angle of insertion in the inferior pole. ^2^^,^^3^ Due to this, some authors recommend use of the trans-subscapularis portal (5-o’clock position), which allows for optimal angulation during the insertion of said anchor. Another reason that popularized the placement of anchors through the 3-o’clock portal is the proximity to important vasculonervous structures in the anteroinferior shoulder face. ^2^ However, the orthogonal placement of the anchor in relation to the glenoid rim promotes greater tensile strength. ^3^^,^^4^

Recent studies in cadavers show that orthogonal anchor insertion has greater resistance to avulsion.

 Number and position of the anchors placed for the reinsertion of the capsulolabral complex are also fundamental for the success of the surgery. ^5^ The anchor placed between the 5- and 6-o’clock positions is the most important for restoration of the anatomical stability of the shoulder. ^6^^,^^7^

 Davidson, Tibone and Resch described the trans-subscapularis portal to achieve a more perpendicular anchor placement in relation to the glenoid rim, in the anteroinferior quadrant of the glenoid. While Davidson and Tibone ^3^ described the “inside out” technique, Resch described the “outside in” technique. However, they recommend the use of the 5-o’clock portal due to the possibility of cephalic vein injury and/or chondral injury to the humeral head. ^3^^,^^8^^,^^9^

This study seeks to radiographically compare anchor positioning in arthroscopic treatment of shoulder instability in both the 3- and the 5-o’clock portals.

## MATERIALS AND METHODS

We retrospectively evaluated 36 patients submitted to arthroscopic reinsertion of the capsulolabral ligament complex to the glenoid by two assistant surgeons from the field, from January 2013 to January 2018. The allocation criteria for each patient varied by surgeon, and each professional used only one of the techniques. A total of 12 patients were operated by the conventional technique of reinsertion of the capsulolabral complex through the portal above the subscapularis tendon (3-o’clock portal) and 24 through the trans-subscapularis (5-o’clock portal). The study was approved by the Ethics Committee, available on Plataforma Brasil by protocol number CAAE: 10008312.7.0000.512

All patients were operated in the beach-chair position, under interscalene brachial plexus block and general anesthesia. After general asepsis, the shoulder to undergo surgery as well as the ipsilateral upper limb were submitted to asepsis and put in a mechanical traction device. A posterior portal 2 cm distal to the posterolateral angle of the acromion was initially created for inspection of the joints; surgery was performed with a 30º arthroscope.

**Figure 1.**
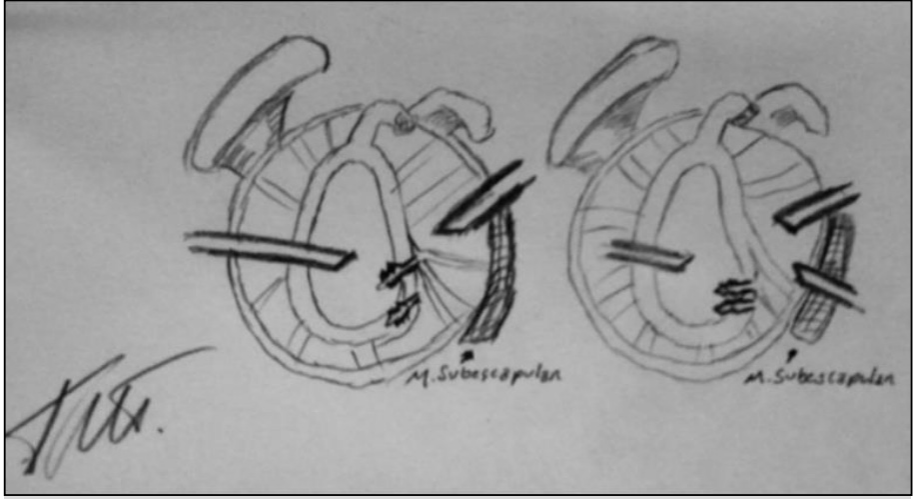
scheme showing the angle of insertion at the 3- and 5-o’clock portals.

**Figure 2.**
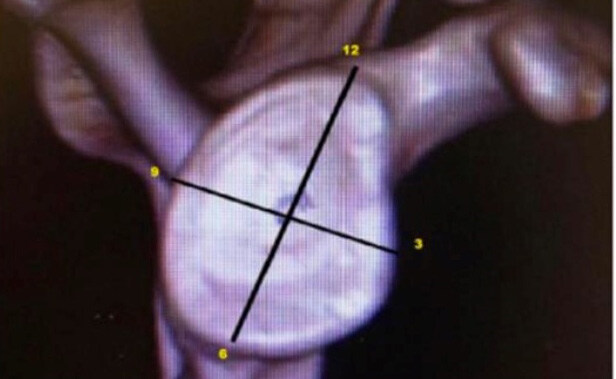
spatial positioning of anchors, how to measure. The upper point is 12-o’clock, the lower one is 6-o’clock, the one on the right is 3 o’clock, and the one on the left is 9-o’clock.

 The two portals for instrumentation (3-o’clock superior to the upper edge of the subscapularis and 5-o’clock at the junction of the middle third with the distal third) ( figure 1 ) ( figure 2 ) were carried out via the “outside in” technique, by verifying the best positioning of the portal with a percutaneous needle and subsequent creation of the portal with a No. 11 scalpel and arthroscopic cannula. 

Then, the glenoid was approached with the initial anchor perforator until the anchor was completely inside the glenoid rim. The capsuloligamenty repair followed the placement of one anchor for each 1 cm of injury, with mattress-type suture following the repair. The patient was then immobilized with a VEUPEAL arm sling for 6 weeks. In the first postoperative week, a true anteroposterior X-ray of the operated shoulder was performed, and the stitches were then removed.

All care before, during and after the procedure was identical between the groups, the only difference being the portal of insertion.

**Figure 3.**
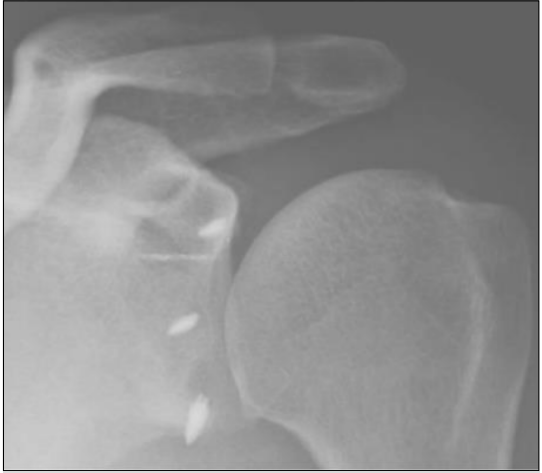
True anteroposterior radiograph showing anchor positioning.

**Figure 4.**
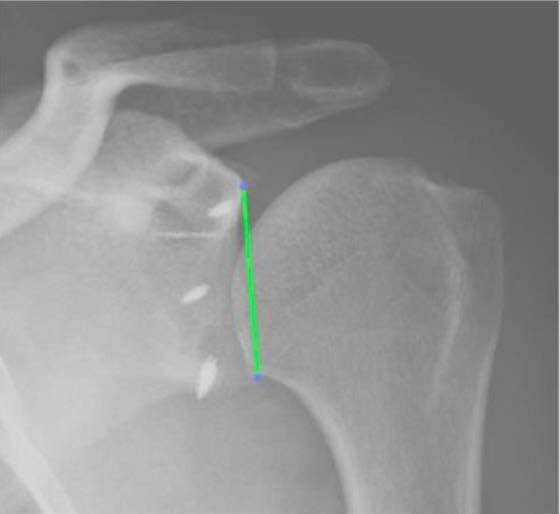
establishment of the long axis of the glenoid.

**Figure 5.**
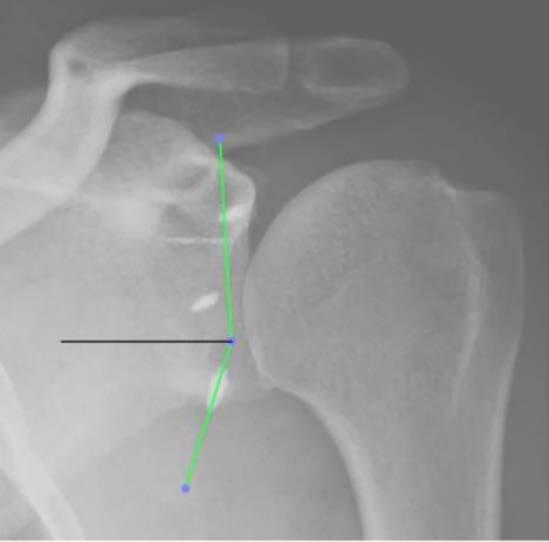
measurement of the angle with the greatest axis of the anchor. Angle = 69.4º

 The radiographic assessment of anchor ( figure 3 ) positioning was performed by a single physician, who was previously trained and did not know the method used. Two measurements were made: 

 The angulation between the supero-inferior axis of the glenoid ( figure 4 ) and the greatest axis of the anchor which we considered the angle of anchor insertion ( figure 5 ). 

**Figure 6.**
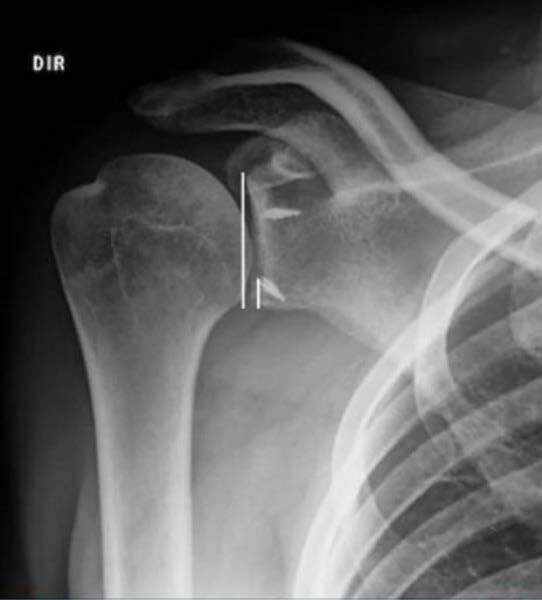
percentage measurement of anchor positioning.

 The distance between the anchor head and the lower pole of the glenoid measured as a percentage in relation to the same supero-inferior axis of the glenoid ( figure 6 ) was taken to eliminate failures due to changes in distortions arising on radiographs (example: ampoule distance). 

Anchors that were partially or completely outside the bone were considered lost. All measurements were performed by a single assistant physician, who was not aware of the method used at the time of surgery.

The measurements were taken via the OsiriX v5. 7 – 32 Bit Switzerland software and for the statistical evaluation we used the GraphPad Prism 7 for Mac USA software. We used ordinal variables for Student’s t-test and categorical variables for Fischer’s test; and considered a significance of p < 0.05.

## RESULTS

We observed 12 patients from the 3-o’clock portal (Group 1) and 24 from the 5-o’clock portal (Group 2).

In Group 1, there was 11 male patients and one female with mean age of 28.08 years; in Group 2, 22 were male and two were female while the mean age was 29.52 years. This makes the two groups homogeneous.

The comparison of the angles means between Group 1 and 2 of the first anchor presented p < 0.0001, while values for the second anchor were p = 0.0005 and for the third anchor p = 0.0019. The comparison of the means of the distance between Groups 1 and 2 in the first anchor presented a significance of p = 0.0189, while for the second anchor it was p = 0.1265 and for the third anchor p = 0.7007.

Regarding the number of anchors lost, the comparison between the groups presented p = 0.4407.

## DISCUSSION

Due to technical and material advances in the arthroscopic treatment of instability, arthroscopic Bankart repair has become widespread and has approached the previous gold standard, that is, open repair.

 The arthroscopic approach to the anteroinferior aspect of the glenoid rim is decisive for the success in the treatment of instability due to the positioning of the cannula, which allows an optimal angle of insertion to the anteroinferior edge of the glenoid. ^10^^-^^13^ In agreement with Dwyer et al., ^14^ we observed that the 5-o’clock portal method is safe, reproducible and allows better anchor positioning when compared to its 3-o’clock counterpart. The present study corroborates this assertion, as it was observed that in the group operated by the 5-o’clock portal the anchors were positioned more orthogonally, which implies a mechanical advantage in avulsion resistance. ^15^

 Khan et al. ^15^ proved that the penetration of the subscapularis by a 5 mm anchor or 8 mm cannula does not produce a deleterious effect on the tendon and it is safe, respecting the distance from the neurovascular structures. As this method does not harm the tendon like in open surgery, there is a potential advantage of fewer complications related to the integrity of the subscapularis tendon. ^14^^,^^16^

 Khan et al. ^15^ also have proven that the use of portals through the rotator cuff does not cause significant anatomical and/or functional injuries to the patient. ^16^ Many variables are currently studied as a prognostic factor for the success of surgical treatment without, however, consensus on the individual value of each variable, such as positioning, type and number of anchors, formation of a “new flap” with mechanical use in shoulder stabilization, restoration of the proprioceptive reflex of the joint, and postoperative immobilization time. ^5^^-^^7^ As well as the association between the positioning of the portals. ^12^^,^^14^^-^^16^ This study presents as strengths the reproducibility of the measurements, the homogeneity of the groups and the fact that it was evaluated by a single physician unaware of the type of procedure performed. One of this study’s weakness was the fact that it was not randomized. 

## CONCLUSION

The results showed that the use of the trans-subscapularis portal (5-o’clock position) was safe with respect to neurovascular structures and improves the anchor positioning in the anteroinferior aspect of the glenoid when compared to the anteroinferior portal (3-o’clock position), allowing a more orthogonal placement of the same.
